# Streptozotocin-Induced Diabetes Decreases Mammary Gland Lipoprotein Lipase Activity and Messenger Ribonucleic Acid in Pregnant and Nonpregnant Rats

**DOI:** 10.1080/15604280212524

**Published:** 2002

**Authors:** Laura Blanco-Dolado, Antonia Martín-Hidalgo, Emilio Herrera

**Affiliations:** 1 Servicio de Bioquímica-Investigación Hospital Ramón y Cajal Madrid Spain; 2 Facultad de Ciencias Experimentales y Técnicas Universidad San Pablo-CEU Boadilla del Monte (Madrid) E-28668 Spain

## Abstract

Diabetes mellitus is associated with a reduction of lipoprotein lipase (LPL) activity in adipose tissue and development of hypertriglyceridemia. To determine how a condition of severe insulin deficiency affects mammary gland LPL activity and mRNA expression during late pregnancy, streptozotocin (STZ) treated (40 mg/kg) and non-treated (control) virgin and 20 day pregnant rats were studied. In control rats, both LPL activity and mRNA were higher in pregnant than in virgin rats. When compared to control rats, STZ-treated rats, either pregnant or virgin, showed decreased LPL activity and mRNA content. Furthermore, mammary gland LPL activity was linearly correlated with mRNA content, and either variable was linearly correlated with plasma insulin levels. Thus, insulin deficiency impairs the expression of LPL in mammary glands, revealing the role of insulin as a modulator of the enzyme at the mRNA expression level.


*Int. Jnl. Experimental Diab. Res.*, 3:61-68, 2002
© 2002 Taylor and Francis
1560-4284/02 $12.00 + .00

### Streptozotocin-induced Diabetes Decreases Mammary Gland Lipoprotein Lipase Activity and Messenger Ribonucleic Acid in Pregnant and Nonpregnant Rats

LAURA BLANCO-DOLADO[1], ANTONIA MARTÍN-HIDALGO[1] AND EMILIO HERRERA[2*]

[1]Servicio de Bioquímica-Investigación, Hospital Ramón y Cajal, Madrid
[2]Facultad de Ciencias Experimentales y Técnicas, Universidad San Pablo-CEU, E-28668 Boadilla del Monte (Madrid), Spain

*Received: October 4, 2001; Accepted:October 28, 2001*

**Diabetes mellitus is associated with a reduction of lipoprotein lipase (LPL) activity in adipose tissue and development of hypertriglyceridemia. To determine how a condition of severe insulin deficiency affects mammary gland LPL activity and mRNA expression during late pregnancy, streptozotocin (STZ) treated (40 mg/kg) and non-treated (control) virgin and 20 day pregnant rats were studied. In control rats, both LPL activity and mRNA were higher in pregnant than in virgin rats. When compared to control rats, STZ-treated rats, either pregnant or virgin, showed decreased LPL activity and mRNA content. Furthermore, mammary gland LPL activity was linearly correlated with mRNA content, and either variable was linearly correlated with plasma insulin levels. Thus, insulin deficiency impairs the expression of LPL in mammary glands, revealing the role of insulin as a modulator of the enzyme at the mRNA expression level.**

*Keywords:* Streptozotocin diabetes, Lipoprotein lipase, Mammary gland, Pregnancy, mRNA, Rat

### INTRODUCTION

Lipoprotein lipase (LPL) is synthesized in parenchymal cells of most tissues in the body and is transported to the capillary endothelium, where triglycerides in circulating chylomicrons and very-low-density lipoproteins are hydrolyzed, thereby facilitating the uptake of

*Corresponding author: e-mail: eherrera@ceu.es hydrolytic products by the subjacent tissues [1]. White adipose tissue, heart, skeletal muscle and lactating mammary gland have the highest LPL activity among body tissues [2,3]. In the lactating mammary gland, the enzyme appears to be synthesized by stromal cells, presumably adipocytes, and transported to the interstitial space where very high concentrations are found [2].

LPL is subject to complex tissue-specific regulation by dietary and hormonal factors through transcriptional, post-transcriptional and post-translational mechanisms. In adipose tissue, insulin is the main positive modulator of LPL activity [4,5], and the decrease in LPL activity that appears during late pregnancy in this tissue has been associated with the insulin resistance occurring in this situation [6,7]. In contrast to adipose tissue, around parturition LPL activity in mammary glands increases [7] and remains high throughout lactation [8]. Besides enhancing the use of circulating triglycerides for milk synthesis, the high LPL activity in mammary gland during late pregnancy, actively contributes to the decline of maternal hypertriglyceridemia [9].

Both insulin and prolactin have been shown to regulate mammary gland LPL activity during lactation [10]. It has been also reported that during late gestation, despite the well known insulin resistant condition, mammary gland is highly sensitive to insulin [11,12]. We have recently reported that hyperinsulinemia increases both mammary gland LPL activity and mRNA during late pregnancy in the rat [13]. The present work was addressed to study the potential relationship between plasma insulin levels and mammary gland LPL activity and mRNA, in pregnant and non-pregnant animals, under conditions of hyperinsulinemia, as caused by pregnancy, and hypoinsulinemia, as caused by streptozotocin treatment.

### MATERIALS AND METHODS

### *Animals and tissue collection*

Female Wistar rats from our colony, weighing 180-200 g., were housed at 22-24 °C and light cycles from 08:00 to 20:00 h. They had free access to water and chow diet (Purina, Panlab, Barcelona, Spain. Rats were made diabetic by a single i.v. injection of streptozotocin (STZ, 40 mg/kg. body wt) dissolved in 50 mM citrate buffer. Control rats were injected with citrate buffer. Two days after STZ treatment, glucosuria was tested to determine the development of diabetes. Only rats showing a positive response were included in the study and 48 h after the STZ injection, these rats were subjected to a daily s.c. injection of 1.5 U/100 g body wt of ultralente bovine insulin (MC, Novo, Denmark) for 7 days. Half of the rats from either STZ or control group were mated with nondiabetic males. The onset of pregnancy was determined by the presence of spermatozoids in vaginal smears, from which time rats were kept without insulin treatment until day 20 of gestation. STZ-treated and control female virgin rats were studied in parallel. Rats had free access to food and were killed between 10:00 and 11.00 h by decapitation. Blood was collected from the neck wound in ice-chilled heparinized tubes for immediate separation of plasma at 4 °C. Mammary glands were rapidly dissected and placed into liquid nitrogen to be stored at -80 °C until processed. Plasma aliquots were used to measure glucose [14], insulin using a specific RIA kit for rats [15] (Novo, Denmark) and triglycerides (Menatest, Italy). The experimental protocol was approved by the Animal Research Committee of the Hospital Ramon y Cajal, Madrid, Spain.

### *RNA preparation*

Total cellular RNA was isolated from frozen rat mammary gland by a single-step acid guanidium thiocyanate-phenol-chloroform extrac-

TABLE I Body weight and plasma components in streptozotocin-diabetic and control pregnant (20 days) and virgin rats

| | Virgin | | Pregnant | |
|---|---|---|---|---|
| | Control | Diabetic | Control | Diabetic |
| Net body weight (g) | 231.2±2.8[a] | 172.4±4.1[b] | 259.8±5.6[c] | 217.2±5.3[a] |
| Glucose (mg/dl) | 131.7±4.1[a] | 682.5±12.5[b] | 90.5±1.6[c] | 449.9±16.8[d] |
| Insulin (μU/ml) | 24.8±1.9[a] | 11.9±1.6[b] | 43.4±5.6[c] | 14.7±1.9[b] |
| Triglycerides (mg/dl) | 97.1±9.7[a] | 405.2±35.9[b] | 322.5±30.8[b] | 599.2±70.5[c] |

N= 11-25 rats / group.
Statistical differences are shown by superscript letters: Different letters express significant statistical differences between the groups tion method [16], using a commercial kit (Ultraspect RNA, Biotecx, USA). Briefly, tissues were homogenized with a polytron in the presence of homogenization buffer. RNA was purified via a series of ethanol precipitations and quantified by optical density at 260 nm.

### *NORTHERN ANALYSIS*

Equal amounts (10 μg) of total RNA were fractionated on 1% agarose gels containing 2.2 M formaldehyde. Electrophoresis was carried out for 18 h at 50 V in 3-(N-morpholino) propanesulfonic acid, pH 7.0, running buffer. RNA was transferred to nylon membrane (Hybond-N+, Amersham, U.K.) for 1 h in 3 M NaCl, 0.3 M sodium citrate, pH 7.0, by a capillarity system and immobilized by cross-linking with ultraviolet light [17]. Nylon membranes were prehybridized for 1 h at 60ºC in 0.5 M sodium phosphate, pH 7.0, 1 mM EDTA, 7% (wt/vol) sodium dodecyl sulfate (SDS), and 1% (wt/vol) bovine serum albumin. Northern hybridation was performed with denatured $^{32}$P-labeled cDNA probes (1 x $10^6$ cpm/ml) for 17-18 h at 60ºC in the same buffer as above. cDNA probe (mouse LPL clone ML 2 [18] was radiolabeled as described by Feinberg and Vogelstein [21] by use of an oligolabeling kit (LKB Biotechnology, Pharmacia, Sweden). DNA (25-50 ng) was labeled to a specific activity of 1-2 x $10^6$ dpm.$mg^{-1}$ using ($^{32}$P)deoxycytidine triphosphate (3,000 Ci/mmol, Amersham, U.K.). Northern filters were washed twice (20 min each wash) with 0.3 M NaCl, 30 mM sodium citrate, pH 7.0, 0.1% SDS at room temperature and twice (20 min each wash) with 15 mM NaCl, 1.5 mM sodium citrate, pH 7.0, 0.1% SDS at 60ºC. Autoradiography was performed with a single intensifying screen at -80ºC and quantified by densitometric scanning.

Northern analyses of whole mammary gland RNA were done for each animal, and bands of 3.6 kb corresponding to LPL mRNA were expressed in glands from all the groups. There were no differences in loading, as verified by ethidium bromide staining of the gels. Bands, corresponding to 28S ribosomal RNA (rRNA) were quantified from the photographs of the gels, and these values were used as internal standard. Thus, LPL mRNA is expressed as the ratio: mRNA/28S rRNA

### *LPL ASSAY*

Total LPL activity was measured as previously described [20, 21]. Briefly, tissue samples were homogenized in 0.2 M Tris(hydroxymethyl)aminomethane (Tris)-HCl, pH 8.2, at 4ºC and delipidated with acetone-diethyl ether. Aliquots of the delipidated samples were assayed for LPL activity. Total LPL activity was measured using an egg lecithin-stabilized emulsion of $^{14}$C-fatty acid-labeled triolein as sub-

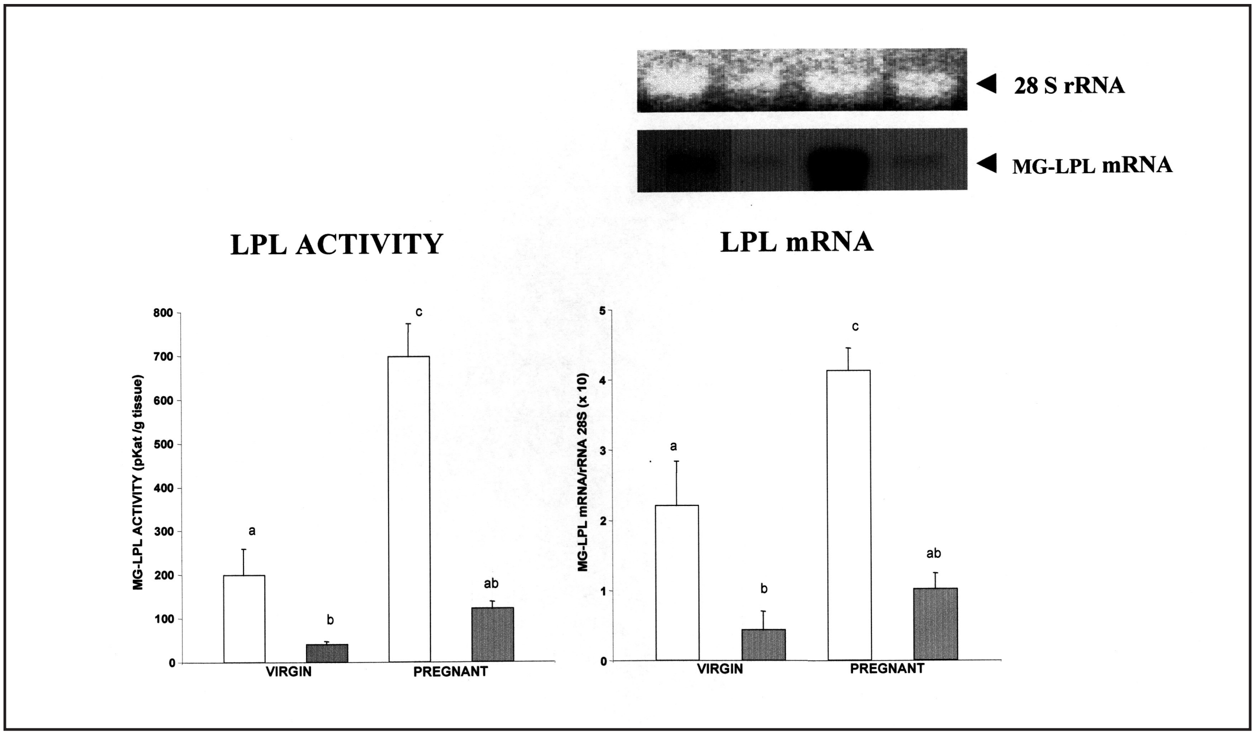

FIGURE 1
Effect of STZ-diabetes on LPL activity and mRNA in mammary gland of virgin and pregnant rats.

Virgin and pregnant STZ-diabetic (*solid bars*) and control rats (*open bars*). Total LPL activity (*left side* of the figure) and LPL mRNA (*right side* of the figure) in mammary gland homogenates were measured as described in *Materials and Methods*. The insert of the figure shows a representative autoradiogram of hybridization of cDNA probes for the LPL mRNA, and a photograph of the gel showing the band corresponding to the 28S rRNA. Statistical comparisons were made by ANOVA, followed by Tukey-Kramer test, with 95% confidence limits. Significance is shown by letters: different letters indicating significant differences between the groups ($p<0.05$). MG= Mammary gland; pkat= pmol of substrate transformed per second.

strate (final concentration 2.5 mM triolein, 2.4% bovine serum albumin, 0.2 M Tris, pH 8.5, 0.1 M NaCl, and 8% heated rat serum in 0.25 mL) in the absence and presence of 1 M NaCl (high saline conditions). LPL activity was determined by subtracting the non-LPL-dependent activity (high salt) from the total lipolytic activity. Data were expressed as pkatals (pmol of substrate transformed per second) per g of tissue.

### *Statistical analysis*

Results are expressed as means ± SE. Statistical comparisons were made with the analysis of variance (ANOVA) followed with Tuckey-Kramer test with 95% confident limits using the Instat Biostatistics program (Graph Pad Instat, Inc., Evanston, IL). Relationship between variables was determined by Pearson correlation coefficient using the Instat program (Instat, Inc., Evanston, IL).

### RESULTS

As shown in Table 1, net body weight (free of conceptus) was higher in pregnant than in virgin, both in control and STZ-diabetic rats, although in the diabetic rats values were always lower than in the respective control rats. Blood glucose levels were highly elevated in diabetic vs. control rats, although values were always lower in pregnant than in virgin rats (Table 1). Although plasma insulin concentration was 2 times higher in control pregnant than in virgin rats, in virgin diabetic rats insulin levels were half than in virgin controls, and did not increase significantly during gestation. Therefore, in comparison to values in their respective controls, hypoinsulinemia in pregnant diabetic rats was much more severe than in virgin diabetic rats. Plasma triglyceride levels were higher in pregnant than in virgin rats. In diabetic rats, plasma triglycerides were higher than in controls, the difference between pregnant and virgin rats remaining significant (Table 1).

The activity of LPL in mammary gland is shown on the left side of Figure 1. LPL activity was 3-4 times higher in control pregnant than in virgin rats. Diabetes caused a greater decrease in mammary gland LPL activity in pregnant than in virgin rats, the difference disappearing between the two groups, indicating a deficient adaptation to pregnancy in the diabetic group. Northern hybridization techniques were employed to explore LPL mRNA expression in mammary gland, and results are illustrated on the right side of Figure 1. The insert of this figure shows a representative autoradiogram of hybridization of cDNA probes for LPL mRNA in the four experimental groups. Mammary gland from control pregnant rats showed higher LPL mRNA than from virgin rats whereas in diabetic rats, a significant decline in the LPL mRNA expression in both pregnant and virgin rats was detected, disappearing the statistical differences between the two groups (right part of Figure 1).

To further investigate the parallel change found in circulating insulin and either mammary gland LPL activity or mRNA expression, linear correlation analysis was performed with all individual values. As shown in Figure 2A, mammary gland LPL activity significantly correlated with plasma insulin levels. To ascertain that such a dependence occurs independently of the reproductive state of the animals, correlations were done including separately virgin and pregnant rats. As expected, LPL activity and

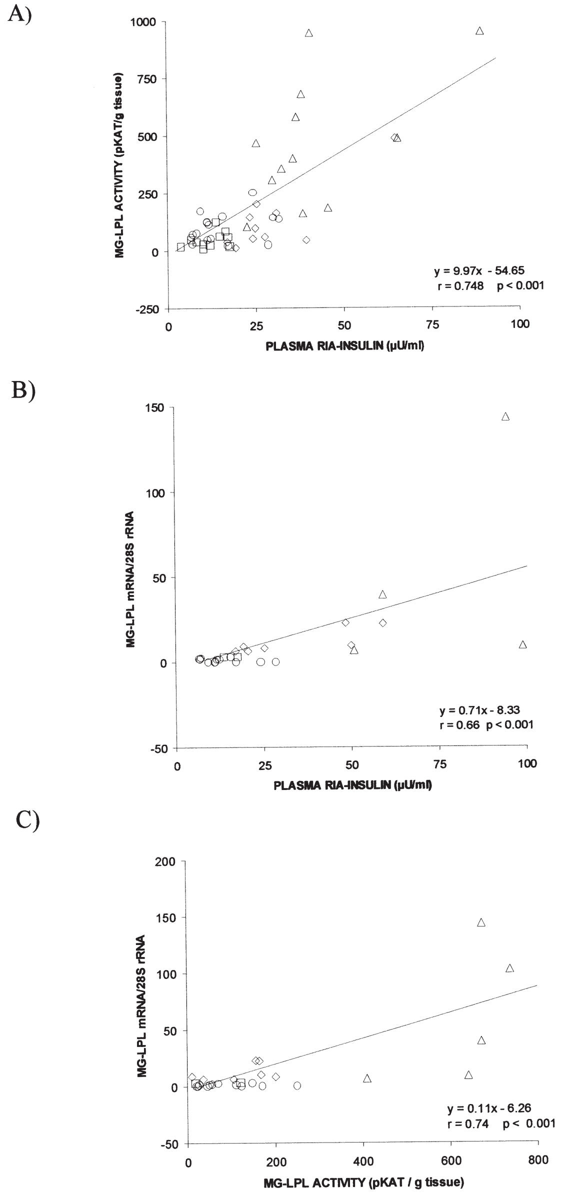

FIGURE 2
Linear correlations for control virgin (◇), STZ-diabetic virgin (□), 20-day pregnant control (△) and STZ-diabetic (○) rats between: A) plasma insulin *versus* total LPL activity in mammary glands; B) plasma insulin *versus* LPL mRNA levels in mammary glands; and C) LPL mRNA *versus* total activity in mammary glands.

plasma insulin correlated significantly in both groups of animals (r =0.81, p<0.001 and r =0.75, p <0.001 in virgin and pregnant rats, respectively). Results in Figure 2B show that LPL mRNA also correlated linearly and significantly with plasma insulin levels. Similarly, significant linear correlations were found when these two variables were compared in virgin or pregnant rats separately (r=0.90, p<0.001 and r=0.67, p<0.001, respectively). As expected from the results presented above and shown in figure 2C, mammary gland LPL activity significantly correlated with its LPL mRNA expression. A significant correlation was also observed when only LPL activity and mRNA values of virgin or pregnant rats were plotted separately (r=0.69, p<0.01 and r=0.75, p<0.001, respectively).

### DISCUSSION

The results presented in this study show that hyperinsulinemia caused by pregnancy enhances mammary gland both LPL activity and mRNA expression, whereas the hypoinsulinemia caused by STZ-diabetes decreases both variables in the gland. Since insulin effects are initiated by the stimulation of the insulin receptor tyrosine kinase after insulin binding, the induction of mammary gland LPL by insulin at late pregnancy agrees with our recent finding that the insulin-stimulated kinase activity of the insulin receptor is augmented in mammary glands of late pregnant rats [11]. This increased LPL activity in mammary glands at the end of gestation, together with the decrease in LPL activity in adipose tissue [22], drive circulating triglyceride-rich lipoproteins to the mammary glands instead of being taken up by adipose tissue for storage [23], contributing actively to the synthesis of milk in preparation for lactation.

LPL is subjected to complex tissue-specific regulation by hormonal factors, which modulate LPL activity via transcriptional, post-transcriptional and post-translational mechanisms. Studies in other tissues, e.g., adipose tissue, have shown that insulin regulates LPL gene expression mainly at the mRNA level [24-26], and accordingly, LPL mRNA content is inversely correlated with the degree of insulin resistance [27]. Furthemore, during late pregnancy, a condition characterized by an impared insulin responsiveness of adipose tissue [28], the decrease in LPL activity in adipose tissue cells has also been shown to parallel changes in mRNA [22]. Since LPL is synthesized in mammary interstitial cells, it is probable, adipocytes [2, 8], the mammary gland LPL, may be regulated similarly to adipose tissue LPL, as previously proposed [29]. However, in contrast to adipose tissue, where LPL activity and mRNA expression are decreased during late pregnancy [22], both variables are enhanced in mammary gland, and this effect could be directly related to the maternal hyperinsulinemia. In fact, when such hyperinsulinemia was prevented by STZ treatment, LPL activity and mRNA content in mammary glands declined in pregnant rats to the values of virgins receiving the same treatment. Besides, changes in LPL activity paralleled those of mRNA content, independently whether the animals were diabetic, control, pregnant or virgin, indicating that the former is controlled at a mRNA level.

Besides hyperinsulinemia and the high insulin sensitivity in mammary glands during pregnancy [11], prolactin may also be involved as a positive mediator of LPL in mammary gland, since its concentration raises around parturition [30] and enhances both LPL activity and mRNA in cultured mammary gland explants from mid-pregnant mice [31]. Although not studied in the diabetic pregnant rat, decreases in plasma prolactin levels have been reported in diabetic pregnant women [32]. Thus, a contribution of decreased prolactin to the low mammary gland LPL activity and mRNA expression in the STZ-diabetic pregnant rats, in addition to their severe hypoinsulinemic condition, cannot be discarded. In fact, another condition of decreased prolactin levels during late pregnancy in rats, caused by progesterone administration, decreases mammary gland LPL activity [9].

Tissue LPL activity depends not only on hormonal regulation but on the physiological status of the animal, changing in response to feeding/fasting, cold adaptation, exercise and tumors [1,33]. In fact, during fasting, mammary gland LPL activity [8], like adipose tissue LPL activity [1, 24, 26], decreases and is subject to regulation at both mRNA and post-translational levels.

In pubertal mice, the predominant cell type in mammary gland is the adipocyte with epithelial structures inter-dispersed among them. With pregnancy, the epithelial structures grow into alveoli that still maintain an intimate association with adipose tissue. During lactation, epithelial cells are the predominant cell types and only small channels of lipid-filled adipocytes could be distinguished because the majority of adipocytes have been depleted of their lipid stores [2]. Thus, mammary gland mass, composition and histology change along pregnancy and lactation [2], and it has been also shown that insulin deficiency decreases the fresh weight of mammary gland by 40% [10]. In any case, STZ diabetes is a complex state and the contribution of other factors that are affecting this condition is possible.

In conclusion, our study shows that hypoinsulinemia contributes to the decreased expression of LPL in mammary glands of diabetic rats, supporting the notion that such effect is caused at the mRNA expression level. Whether the decrease in mRNA is due to an inhibition of LPL gene transcription and/or an increased rate of LPL mRNA degradation remains to be established.

***Acknowlegments***

We acknowledge the excellent technical assistance of Ms. Lydia Huerta. This study was supported by Grant 00/0229 from the Fondo de Investigaciones Sanitarias de la Seguridad Social of Spain. We thank Drs. C. Holm and M.C. Schotz for the gift of mouse LPL cDNA probes.

### REFERENCES

1. Eckel RH (1989) Lipoprotein lipase. A multifunctional enzyme relevant to common metabolic diseases. *N Engl J Med* **320**:1060-1068.
2. Jensen DR, Bessesen DH, Etienne J, Eckel RH, Neville MC (1991) Distribution and source of lipoprotein lipase in mouse mammary gland. *J Lipid Res* **32**:733-742.
3. Blanchette-Mackie EJ (1991) Lipoprotein lipase and fatty acid transport in heart, adipose tissue and mammary gland:immuni- and cytochemistry. *Endocr Regul* **25**:63-69.
4. Oller do Nascimento CM, Ilic V, Williamson DH (1989) Re-examination of the putative roles of insulin and prolactin in the regulation of lipid deposition and lipogenesis *in vivo* in mammary gland and white and brown adipose tissue of lactating rats and litter-removed rats. *Biochem J* **258**:273-278.
5. Oliver JD, Rogers MP (1993) Stimulation of lipoprotein lipase synthesis by refeeding, insulin and dexamethasone. *Biochem J* **292**:525-530.
6. Herrera E, Ramos P, Martín A (1990) Control by insulin of adipose tissue lipoprotein lipase activity during late pregnancy in the rat. In: Shafrir E (ed) Frontiers in Diabetes Research. Lessons from Animal Diabetes III. Smith-Gordon, London, vol XI:551-554.
7. Ramos P, Herrera E (1996) Comparative responsiveness to prolonged hyperinsulinemia between adipose-tissue and mammary-gland lipoprotein lipase activities in pregnant rats. Early Pregnancy: *Biology and Medicine* **2**:29-35.
8. Jensen DR, Gavigan S, Sawicki V, Witsell DL, Eckel RH, Neville MC (1994) Regulation of lipoprotein lipase activity and mRNA in the mammary gland of the lactating mouse. *Biochem J* **298**:321-327.
9. Ramirez I, Llobera M, Herrera E (1983) Circulating triacylglycerols, lipoproteins, and tissue lipoprotein lipase activity in mothers and offspring during the perinatal period: effect of postmaturity. *Metabolism* **32**:333-341.
10. Da Costa THM, Williamson DH (1994) Regulation of rat mammary-gland uptake of orally administered [1-$^{14}$C]triolein by insulin and prolactin: Evidence for bihormonal control of lipoprotein lipase activity. *Biochem J* **300**:257-262.
11. Carrascosa JM, Ramos P, Molero JC, Herrera E (1998) Changes in the kinase activity of the insulin receptor account for the increased insulin sensitivity of the mammary gland in late pregnancy. *Endocrinology* **139**:520-526.

12. Burnol A-F, Ferré P, Leturque A, Girard J (1987) Effect of insulin on in vivo glucose utilization in individual tissues of anesthetized rats. *Am J Physiol* **252**:E183-E188.
13. Ramos P, Martín-Hidalgo A, Herrera E (1999) Insulin-induced up-regulation of lipoprotein lipase messenger ribonucleic acid and activity in mammary gland. *Endocrinology* **140**:1089-1093.
14. Hugget ASG, Nixon DA (1957) Use of glucose oxidase, peroxidase and O-dianisidine in determination of blood and urinary glucose. *Lancet* **1**:368-370.
15. Heding LG (1972) Determination of total serum insulin (IRI) in insulin treated diabetic patients. *Diabetologia* **8**:2260-266.
16. Chomezynski P, Sacchi N (1987) Single/step method of RNA isolation by guanidinium thiocyanate-phenol-chloroform extraction. *Anal Biochem* **162**:156-159.
17. Church GM, Gilbert W (1984) Genomic sequencing. *Proc Natl Acad Sci USA* **81**:1991-1995.
18. Kirchgessner TG, Svenson KL, Lusis AJ, Schotz MC (1987) The sequence of cDNA encoding lipoprotein lipase. *J Biol Chem* **262**:8463-8466.
19. Feinberg AP, Vogelstein B (1983) A technique for radiolabeling DNA restriction endonuclease fragment to high specific activity. *Anal Biochem* **132**:6-13.
20. Llobera M, Montes A, Herrera E (1979) Lipoprotein lipase activity in liver of the rat fetus. *Biochem Biophys Res Commun* **91**:272-277.
21. Nilsson-Ehle P, Schotz MC (1972) A stable radiactive substrate emulsion for assay of lipoprotein lipase. *J Lipid Res* **17**:536-541.
22. Martín-Hidalgo A, Holm C, Belfrage P, Schotz MC, Herrera E (1994) Lipoprotein lipase and hormone-sensitive lipase activity and mRNA in rat adipose tissue during pregnancy. *Am J Physiol* **266**:E930-E935.
23. Argilés J, Herrera E (1989) Appearance of circulating and tissue $^{14}C$-lipids after oral $^{14}C$-tripalmitate administration in the late pregnant rat. *Metabolism* **38**:104-108.
24. Ong JM, Kirchgessner TG, Schotz MC, Kern PA 1988 Insulin increases the synthetic rate and messenger RNA level of lipoprotein lipase in isolated rat adipocytes. *J Biol Chem* **263**:12933-12938.
25. Raynolds MV, Awald PD, Gordon DF, Gutierrez-Hartmann A, Rule DC, Wood WM, Eckel RH (1990) Lipoprotein lipase gene expression in rat adipocytes is regulated by isoproterenol and insulin through different mechanisms. *Mol Endocrinol* **4**:1416-1422.
26. Semb H, Olivecrona T (1989) Two different mechanisms are involved in nutritional regulation of lipoprotein lipase in guinea-pig adipose tissue. *Biochem J* **262**:505-511.
27. Maheux P, Azhar S, Kern PA, Chen Y-DI, Reaven GM (1977) Relationship between insulin-mediated glucose disposal and regulation of plasma and adipose tissue lipoprotein lipase. *Diabetologia* **40**:850-858.
28. Leturque A, Ferré P, Burnol A-F, Kande J, Maulard P, Girard J (1986) Glucose utilization rates and insulin sensitivity *in vivo* in tissues of virgin and pregnant rats. *Diabetes* **35**:172-177.
29. Neville MC, Waxman LJ, Jensen D, Eckel RH (1991) Lipoprotein lipase in human milk: compartmentalization and effect of fasting, insulin, and glucose. *J Lipid Res* **32**:251-257.
30. Henninghausen L, Robinson GW, Wagner K-U, Liu X (1997) Prolactin signaling in mammary gland development. *J Biol Chem* **272**:7567-7569.
31. Hang J, Rillema JA (1997) Prolactin's effects on lipoprotein lipase (LPL) activity and on LPL mRNA levels in cultured mouse mammary gland explants. *Proc Soc Exp Biol Med* **214**:161-166.
32. Montelongo A, Lasunción MA, Herrera E (1992) Longitudinal study of plasma lipoproteins and hormones during pregnancy in normal and diabetic women. *Diabetes* **41**:1651-1659.
33. Eckel RH (1987) Adipose tissue lipoprotein lipase. In: Borensztajn J (ed) Lipoprotein lipase. Evener, Chicago, pp79-132.

